# Social functioning outcomes in men and women receiving medication-assisted treatment for opioid use disorder

**DOI:** 10.1186/s13293-020-00298-4

**Published:** 2020-04-23

**Authors:** Emma A. van Reekum, Tea Rosic, Jacqueline Hudson, Nitika Sanger, David C. Marsh, Andrew Worster, Lehana Thabane, Zainab Samaan

**Affiliations:** 1 Michael G DeGroote School of Medicine, McMaster University, Hamilton, ON Canada; 2 Department of Psychiatry and Behavioural Neurosciences, McMaster University, Hamilton, ON Canada; 3 Northern Ontario School of Medicine, Sudbury, ON Canada; 4 Canadian Addiction Treatment Centres, Markham, ON Canada; 5 Department of Health Research Methods, Evidence, and Impact, McMaster University, Hamilton, ON Canada; 6 Department of Medicine, McMaster University, Hamilton, ON Canada; 7 Biostatistics Unit, Research Institute at St Joseph’s Healthcare, Hamilton, ON Canada; 8 Departments of Pediatrics/Anesthesia, McMaster University, Hamilton, ON Canada

**Keywords:** Opioid use disorder, Social functioning, Methadone, Buprenorphine, Sex differences

## Abstract

**Background:**

Social functioning (SF), the ability to engage with life and fulfill roles may be a salient “patient important outcome” in addiction treatment. It is not known if medication-assisted treatment (MAT) impacts SF in opioid use disorder (OUD). There is a growing evidence to suggest that men and women are impacted differently by OUD. This study is the largest to date to study sex differences in OUD and explore associations between MAT and SF.

**Methods:**

Data were collected from 2736 participants with OUD, enrolled in MAT for varying lengths of time, in outpatient clinics across Ontario. SF was defined according to the Maudsley Addiction Profile’s domains of (1) employment, (2) criminal activity, and (3) interpersonal conflict. Using logistic regression analysis, we examined sociodemographic and clinical factors associated with domains of SF.

**Results:**

There were 1544 men (56%) and 1192 women (44%) in this study, and ages varied from 17 to 76 years for men and 18 to 69 years for women. At study entry, participants had been on MAT for a median of 2 years. Compared to men, women reported more psychological (mean MAP score 14/40, SD = 9.55, versus 11/40, SD = 8.64; *p* < 0.001) and physical symptoms (mean MAP score 17/40, SD = 7.70 versus 14/40, SD = 7.74; *p* < 0.001). More women reported unemployment(74% versus 58%; *p* < 0.0001) and interpersonal conflict (46% versus 35%; *p* < 0.0001). Men were more likely than women to report criminal activity (11%, versus 8%; *p* = 0.001). Psychological symptoms increased the risk of worse SF, across domains, for men and for women. Every year on MAT was associated with a 7% increase in the odds of women engaging with criminal activity (OR = 1.07, 95% CI 1.02, 1.12, *p* = 0.006).

**Conclusions:**

Men and women had different SF profiles and psychological symptoms scores while on MAT. The length of time on MAT increased the risk of criminal activity in women, and overall, duration of MAT was not associated with improvement in SF. This may suggest that MAT alone may not support continual improvements in SF in OUD.

## Background

North America is in the midst of an ongoing “opioid crisis”: a rise in illicit and prescribed opioid use, as well as opioid-related mortality and morbidity [1]. In 2017, the USA saw 70,237 opioid drug overdose deaths: a significant rise from 2016 [2], with a 70% increase in deaths in certain regions [3]. Similar patterns exist in Canada, leading the government to declare the opioid crisis a public health emergency [4]. Opioids negatively impact people of all demographics; however, men account for 75% of deaths, and those aged 30–39 appear to be particularly affected [3, 5, 6]. There is a paucity of data on women who use opioids, as women are generally underrepresented in addiction research [1, 7].

Opioid use can lead to a myriad of other harms beyond overdose and mortality including increased transmission of communicable disease [8], risky behavior (e.g., driving under the influence) [9], economic burden [10], and comorbid mental disorders [11]. Social functioning (SF), the ability to successfully engage with life and to fulfill personal roles (e.g., vocational, societal, interpersonal), may also be impacted by opioid use. Indeed, impaired SF is a hallmark criterion in the diagnosis of opioid use disorder (OUD), and considered one of the most debilitating features of the disease [12]. Despite SF being an important diagnostic feature, there is a dearth of research addressing SF in OUD. Only one trial to date, a small (*n* = 164) sampled Australian study, specifically examined SF in opioid users; data showed that, compared with matched controls, SF was significantly lower in patients receiving treatment for opioid use [13]. Studies involving other psychiatric disorders have linked impaired SF to greater drug use and depressive symptomatology [14]. A meta-analysis, involving studies conducted before the opioid crisis, found that domains of SF, specifically family support and engaging in house work, predicted retention in medication-assisted treatment (MAT) for OUD [15].

MAT is first line for treating OUD [16, 17]. Methadone or buprenorphine-naloxone are used to thwart craving and withdrawal, and minimize harms associated with opioids [17]. Given buprenorphine-naloxone’s safety profile, and significant risks associated with methadone (e.g., fatal overdose, prolongation of the QTc), the most recent guidelines recommend this medication as initial treatment rather than methadone [17]. These medications are recommended with or without supportive treatments like psychosocial interventions and contingency management [16]

Benefits of MAT are well established in the literature, including reduced all-cause and overdose-related mortality [18], moderated illicit opioid use [19], and improved quality of life [20, 21]. Furthermore, as Nosyk et al. found that the costs of criminal activity were lower while participants were enrolled in MAT (either injectable diacetylmorphine or methadone) compared with relapse states [22], MAT may also precipitate reductions in criminal behavior. A small (*n* = 102) study from 1998 assessed self-report quality of life in people with OUD prior to, and one year after initiating methadone. In those who remained in treatment (*n* = 48), significant improvements were reported in multiple quality of life domains, including close relationships, leisure activities, and physical abilities [20]. The NAOMI randomized controlled trial examined injectable diacetylmorphine and methadone for 251 patients with long-standing opioid use [21]. This trial, that was conducted prior to the initiation of the opioid epidemic, found that the majority of participants (*n* = 132) described improvements in health-related quality of life after 12 months of treatment.

The differences between men, women, and gender-diverse people are boundless, straddling biological, psychological, and sociological realms [23]. From a biological lens, for example, men and women differ in terms of pharmacokinetics and response to treatments [24], as well as cardiovascular disease onset, risk, and clinical presentation [25]. People of different gender identities also function differently in, and fulfill diverse roles in society; indeed, “gender norms” are considered a determinant of health shared by all people [26]. For these reasons, the importance of incorporating sex and/or gender analyses into clinical research and guidelines is increasingly recognized and promoted.

Furthermore, a 2015 meta-analysis of small sampled studies found that women are more likely to use illicit amphetamines, experience unemployment, and report criminal activity during OUD treatment [27]. This meta-analysis only included participants on methadone, and acknowledged a high risk of bias in most studies (attributed to limited sample size, poor outcome measures, and not adjusting for confounders); authors called for additional high-quality studies to better characterize sex differences in OUD treatment. Since then, and despite well-established sex differences in OUD, a recent systematic review of buprenorphine-naloxone found that just half of included studies conducted sex-specific analyses. Further, women represented just one quarter of participants enrolled in randomized controlled trials for MAT [28]; this, despite higher past-year prevalence of prescription opioids in Canadian women [29] and higher rates of illicit opioids in other countries [1]. Authors of this meta-analysis called for greater inclusion of women in addictions research as well as future trials to address sex-specific outcomes in MAT, and in particular, for the first-line medication, buprenorphine-naloxone [28].

It is presently not known whether MAT impacts SF in OUD. Additionally, a full picture of the SF of North American men and women in treatment for OUD has not been determined. In particular, a gap in the literature exists in understanding sex differences of SF in OUD and whether MAT impacts SF of men and women in treatment. This study will address recommendations/limitations in the literature as determined by prior meta-analyses and reviews [27, 28]. Specifically, we aim to

1. Examine sex differences in demographic and clinical characteristics in a cohort of patients receiving MAT for OUD.
2. Assess the SF of men and women in this cohort, as measured by employment, interpersonal conflict, and criminal activity.
3. Assess whether MAT is associated with improved SF.
4. Explore demographic and clinical characteristics associated with SF.

## Methods

### Data

Data were employed from two large sampled, prospective, cohort studies: The GENetics of Opioid Addiction (GENOA) study and the Pharmacogenetics of Opioid Substitution Treatment (POST) study. Between 2011 and 2017, data for the GENOA study were collected from 1390 people receiving MAT in outpatient clinics. All participants were at least 18 years old, met diagnostic criteria for an OUD as per the Diagnostic and Statistical Manual of Mental Disorders (DSM IV) [30] and were receiving MAT for varying durations, in order to treat their OUD. Considerable data were collected at study entry, including self-report demographic, medical history, and MAT (e.g., dose, duration of treatment) information. Participants also underwent a structured clinical interview, administered by trained research staff, and provided urine and blood samples. Please see previous literature for a more thorough description of GENOA methodology [31, 32]. The POST study began in 2018 as an extension to GENOA, with similar data collection protocol and inclusion criteria, barring participants now meeting criteria for OUD as diagnosed with the DSM-5 [12]. The POST study is ongoing; as of June 2019, data were collected from 1769 participants.

Participants from both GENOA and POST were recruited from MAT centres in Ontario, Canada. These outpatient clinics are centrally managed through the Canadian Addiction Treatment Centres (CATC), and thus, observe identical treatment protocols. All participants provided written consent prior to study involvement. All study procedures comply with the ethical standards for human experimentation; ethics approval was obtained from the Hamilton Integrated Research Ethics Board (GENOA project ID 11-056; POST project ID 4556).

**Fig. 1:**
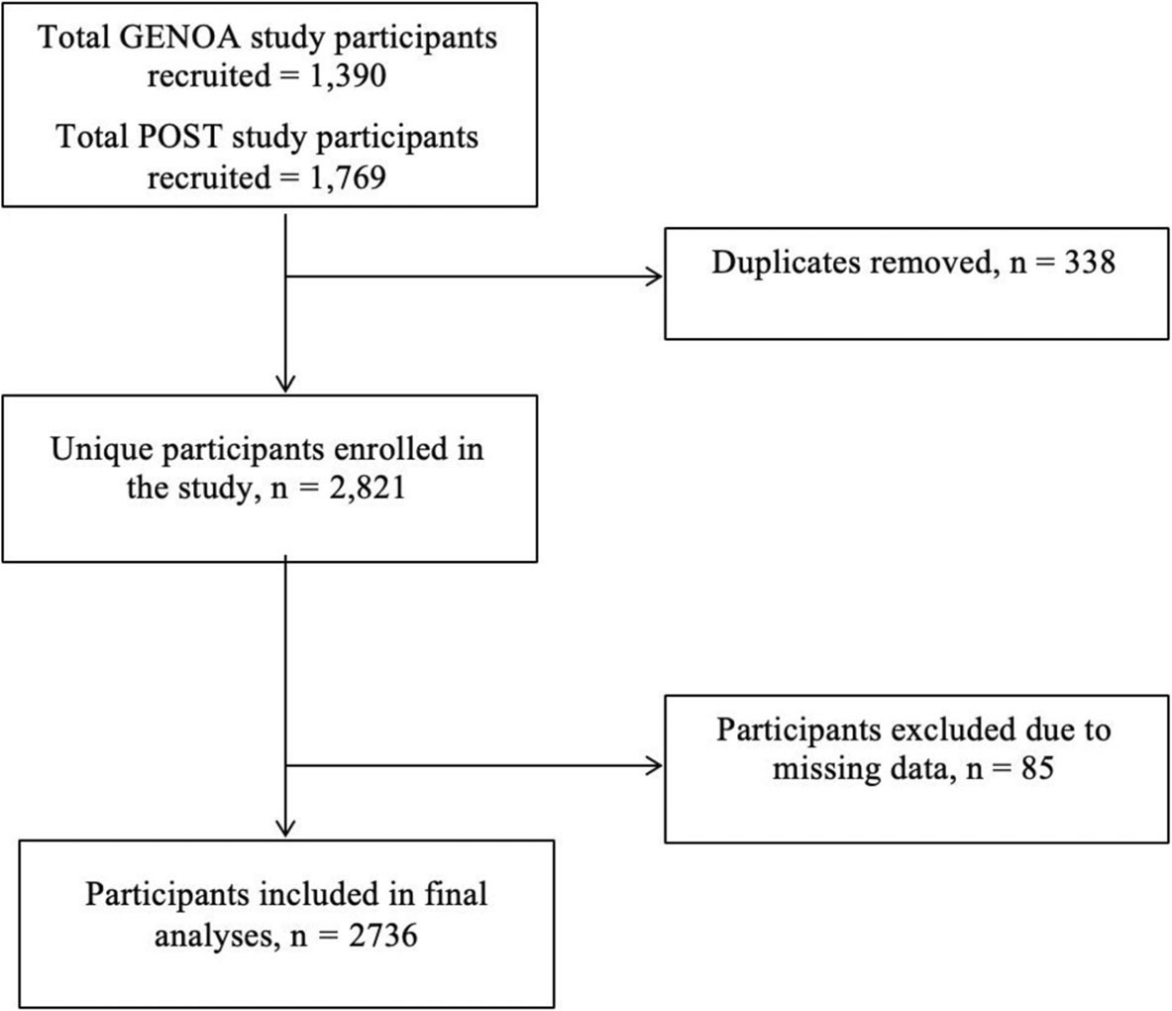
Study flow diagram. Illustration of participant recruitment, duplication, enrolment, exclusion, and final analyses

Data were merged from GENOA and POST for the present study. Duplicate enrolment were removed leaving unique individual participants. When duplication was identified, the GENOA data were retained, and the POST data were removed. As shown in Fig. 1, duplicate enrolment impacted 339 participants. Further data exclusion (*n* = 85) occurred for participants with missing data from the urine collection, from the main outcome measure, the Maudsley Addiction Profile (MAP), or if the participant was transferred to another clinic for treatment. Reporting of this study was guided by recommendations from the Strengthening the Reporting of Observational Studies in Epidemiology (STROBE) guidelines [33].

### Study instruments and procedures

One of the study objectives was to determine if MAT is associated with SF, our primary outcome. As participants had been in MAT for varying lengths of time at study entry, we examined for associations between duration of treatment and SF. Assessment of treatment outcome was conducted using the Maudsley Addiction Profile (MAP) [34]. This is a well-validated and commonly employed measure, used to assess for past-month physical and psychological symptoms as well as SF in those receiving treatment for substance use. The MAP considers SF to be an amalgamation of three domains: (1) employment; (2) criminal activity; and (3) interpersonal conflict. Employment is assessed via three questions, however, for simplicity, this study used a dichotomous “yes/no” variable for employment. Criminal activity was obtained by asking respondents to describe on how many days, and the number of times on a typical day, certain crimes (selling drugs, fraud, theft, shoplifting, and other) were committed. From this information, a dichotomous variable was created for the reporting of any criminal activity, “yes/no,” and a continuous variable denotes the number of episodes of crime per month, in those who reported any criminal activity. In order to discern interpersonal functioning, the MAP asks how many days respondents have contact (face-to-face or telephone) with partners, relatives, and friends, as well as on how many days there was serious conflict with those people. Conflict was considered “serious” if it involved a major argument, verbal abuse, or violence. As with criminal behavior, a dichotomous variable was created for the reporting of any conflict, “yes/no,” and a continuous variable describes the percentage of contact time spent in conflict, for those who experienced conflict. All participants received a face-to-face interview, as per MAP protocol, and data were entered into the Research Electronic Data Capture (REDCap) [35] tool.

As data were cross-sectional, this objective is purely associative in nature, and causality should not be implied. However, we controlled for confounders including (a) age; (b) type of MAT (methadone vs. buprenorphine-naloxone); (c) dose of MAT; (d) illicit opioid use; (e) physical symptoms; and (f) psychological symptoms. All analyses were conducted by sex. Illicit drug use was garnered from routine weekly/biweekly urine drug screens, collected in keeping with standard protocol at CATC sites. Urine was collected and analyzed to determine non-methadone or non-buprenorphine opioid use, using the IMDx^TM^ Prep assay. The percentage of opioid-positive urine screens for each participant was computed. Physical and psychological symptoms were each derived from 10 distinct items on the MAP [34]. Physical symptoms of “poor appetite,” “tiredness,” “nausea,” “stomach pains,” “difficulty breathing,” “chest pains,” “joint/bone pains,” “muscle pains,” “numbness/tingling,” and “tremor” were assessed on a 5-point Likert scale. Similarly, a 5-point Likert scale was employed for transdiagnostic symptoms of anxiety and depression: “feeling tense,” “scared,” “fearful,” “nervousness,” “panic,” “hopelessness,” “worthlessness,” “anhedonia,” and “loneliness.” Items for both physical and psychological symptoms were summed, giving a total symptom score from 0 to 40, with 40 signifying the most severe symptomatology.

### Statistical analysis

All statistical tests were conducted using STATA version 15.1 (StataCorp LP, College Station, TX, USA). Our first and second objectives were to examine sex differences in MAT. As such, demographic characteristics and descriptive statistics are presented by sex, assessed using independent *t* tests for normally distributed data and the Mann-Whitney *U* and Wilcoxon rank-sum test where data were skewed (Table 1). For continuous variables, mean values and standard deviations (SDs) are presented for normal distributions, and median and interquartile ranges (IQRs) are reported for skewed distributions. Percentages were tabulated for categorical data.

**Table 1:** Baseline demographic and clinical characteristics, by sex (*n* = 2736)

Characteristic	Women	Men	*p*
Sex (*n*, % of total)	1192, 44%	1544, 56%	
Age in years (mean ± SD, min–max)	37.58 ± 10.64, 18–69	39.12 ± 11.14, 17–76	< 0.001***
Type of MAT (*n*, % of total on Methadone)	1008, 85%	1313, 85%	0.731
Type of MAT (*n*, % of total on Buprenorphine)	184, 15%	231, 15%	0.731
Methadone dose; mg/day (mean ± SD, min–max)	68.30 ± 40.13, 0–320	73.23 ± 45.05, 2–405	0.006**
Buprenorphine dose; mg/day (mean ± SD, min–max)	12.31 ± 7.24, 1–40	11.82 ± 6.74, 0–32	0.478
Years on MAT (median, IQR, min–max)	2, 4.71, 0–40	2, 4.42, 0–33	0.432
New to MAT; < 1 year (*n*, % of total)	436, 43%	564, 43%	0.904
Percentage of opioid-positive urine drug screens (mean ± SD)	18.15% ± 26.49	19.42% ± 27.88	0.228
Physical symptoms (mean score ± SD)	16.50 ± 7.70	13.62 ± 7.74	< 0.001***
Psychological symptoms (mean score ± SD)	14.01 ± 9.55	11.37 ± 8.64	< 0.001***
Unemployed (*n*, % of total)	882, 74%	902, 58%	< 0.001***
Any crime (*n*, % of total)	92, 8%	177, 11%	0.001**
Episodes of crime per month, in those who commit crime (median, IQR)	4, 25	9, 88	0.032*
Any conflict (*n*, % of total)	550, 46%	533, 35%	< 0.001***
Percentage of contact time spent in conflict, in those reporting conflict (median, IQR)	8.45%, 26.10%	7.69%, 20.20%	0.596

*p < 0.05

**p < 0.01

***p < 0.001

Min–max = minimum–maximum, *MAT* = medication-assisted treatment, *SD* = standard deviation, *IQR* = interquartile range

Our final objectives were to explore associations between MAT and the three domains of SF as captured by the MAP. We constructed three logistic regression models, with the dichotomous variables of employment, crime, and interpersonal conflict as dependent variables. Length of time in MAT was the main covariate of interest in the regression models, as we aimed to explore the association between time in treatment and SF outcomes. Additional covariates included in the models were (a) age; (b) type of MAT; (c) dose of MAT; (d) illicit opioid use; and (e) physical and (f) psychological symptoms. Selection of covariates was guided by prior research suggesting potential influence on domains of SF: psychological distress is consistently associated with unemployment [36, 37]; relationship satisfaction has been shown to increase with age [38]; and the relationship between age and crime (the “age-crime curve”) is well established in the literature, wherein crime tends to peak in adolescence and decrease thereafter [39]. Results from the logistic regression analyses are presented as odds ratios (OR) with 95% confidence intervals (CI). This study employed a level of significance of 0.05 for hypothesis testing. Given a sample size of 2736, this study is sufficiently powered to conduct all described analysis (e.g., 10 participants per covariate in the regression model) [40].

## Results

### Participant characteristics

A total of 2736 participants were included in the analyses (Fig. 1), and 56% were men. Participants varied in age from 17 to 76 years (Table 1). Men were older than women (mean age 39.12 years, SD = 11.14, versus 37.6 years, SD = 10.6, *p* < 0.001).

Participants had been in MAT from 0 to 40 years, with a median of 2 years (IQR = 4.42); there was no treatment duration difference between men and women (*p* = 0.478) (Table 1). The majority of participants were on methadone (85%), as opposed to buprenorphine-naloxone (15%), with no sex differences observed (*p* = 0.731). Men in this study were on significantly higher doses of methadone (mean dose 73.23 mg/day, SD = 45.05, versus 68.30 mg/day, SD = 40.13; *p* = 0.006), but on comparable doses of buprenorphine-naloxone (mean dose 11.82 mg/day, SD = 6.74, versus 12.31 mg/day, SD = 7.24; *p* = 0.478).

Women self-reported significantly more physical symptoms than men (mean MAP physical symptom score 17/40, SD = 7.70 versus 14/40, SD = 7.74; *p* < 0.001). Women also self-reported significantly more psychological symptoms (mean MAP psychological symptom score 14/40, SD = 9.55 versus 11/40, SD = 8.64; *p* < 0.001; Table 1).

### Social functioning

We assessed sex differences in domains of SF, including unemployment, interpersonal conflict, and criminal activity (Table 1). Women reported a higher rate of unemployment (74% versus 58%; *p* < 0.0001; Table 1). Women were also more likely to report interpersonal conflict (46% versus 35%; *p* < 0.0001); however, men and women who reported conflict endorsed similar amounts of contact time spent in conflict (8% versus 8%; *p* = 0.596). Men were more likely than women to report criminal activity (11%, versus 8%; *p* = 0.001). For those participants who reported any criminal activity, men committed significantly more crimes per month than women (median = 9, IQR = 88, versus median = 4, IQR = 25; *p* = 0.032), with some men reporting up to 100 episodes of illicit drug sales per day, totalling 3000 crimes in a month.

Post hoc subgroup analyses were conducted to explore differences between participants who recently initiated MAT (i.e., 1 year or less) and those on MAT for longer durations (i.e., greater than one year). Treatment duration did not impact employment for men or women, nor did it impact crime or conflict for women. However, men who initiated MAT within one year were more likely to report crime (15% versus 10%, *p* = 0.004) and conflict (38% versus 33%, *p* = 0.032) than men who were on MAT for longer.

### Factors associated with social functioning

In Tables 2, 3, and 4, we present the results of logistic regression models examining participants’ characteristics associated with employment status, criminal activity, and interpersonal conflict for men and women. Older age significantly decreased the odds of employment for both men and women (OR = 0.98, 95% CI 0.97, 0.99; OR = 0.97, 95% CI 0.95, 0.98; respectively; Table 2). Men and women who endorsed more psychological symptoms were less likely to be employed (OR = 0.96, 95% CI 0.94, .097; OR = 0.96, 95% CI 0.95, 0.98; respectively). Men on higher doses of methadone or buprenorphine-naloxone were less likely to be employed (OR = 0.996, 95% CI 0.99, 0.999). Length of time in MAT was not associated with employment status (OR = 0.98, 95% CI 0.95, 1.01; OR = 0.97, 95% CI 0.95, 1.00).

**Table 2:** Multivariable model of factors associated with employment status, by sex (*n* = 2736)

	For men				For women			
Covariate	OR	95% CI		*p*	OR	95% CI		*p*
Age (years)	.978	.968	.990	< 0.001***	.968	.954	.982	< 0.001***
MAT type	.826	.590	1.16	0.286	1.06	.700	1.60	0.786
MAT dose (mg/day)	.996	.993	.999	0.003**	.997	.993	1.00	0.140
Years on MAT	.980	.952	1.01	0.155	.965	.923	1.00	0.060
Opioid-positive urine drug screens (% of total)	.998	.994	1.00	0.184	.998	.992	1.00	0.384
Physical symptoms	.986	.970	1.00	0.098	.993	.972	1.01	0.520
Psychological symptoms	.958	.944	.973	< 0.001***	.962	.945	.980	< 0.001***

**Table 3:** Multivariable model of factors associated with crime, by sex (*n* = 2736)

	For men				For women			
Covariate	OR	95% CI		*p*	OR	95% CI		*p*
Age (years)	.971	.955	.988	0.001***	.958	.934	.984	0.001**
MAT type	.984	.579	1.67	0.951	1.42	.726	2.76	0.308
MAT dose (mg/day)	1.00	.995	1.00	0.875	.995	.989	1.00	0.1142
Years on MAT	.965	.917	1.02	0.171	1.07	1.02	1.12	0.006**
Opioid-positive urine drug screens (% of total)	1.01	1.01	1.02	< 0.001***	1.01	1.00	1.02	0.007**
Physical symptoms	1.00	.979	1.03	0.784	.967	.934	1.00	0.062
Psychological symptoms	1.05	1.03	1.08	< 0.001***	1.06	1.03	1.09	< 0.001***

**Table 4:** Multivariable model of factors associated with conflict, by sex (*n* = 2736)

	For men				For women			
Covariate	OR	95% CI		*p*	OR	95% CI		*p*
Age (years)	.964	.953	.975	< 0.001***	.966	.954	.979	< 0.001***
MAT type	.741	.514	1.07	0.109	.830	.566	1.22	0.340
MAT dose (mg/day)	.999	.996	1.00	0.449	.996	.993	1.00	0.025*
Years on MAT	1.00	.972	1.03	0.928	1.00	.977	1.03	0.768
Opioid-positive urine drug screens (% of total)	1.01	1.00	1.01	< 0.001***	.998	.994	1.00	0.429
Physical symptoms	1.05	1.03	1.06	< 0.001***	1.02	1.00	1.04	0.051
Psychological symptoms	1.03	1.02	1.05	< 0.001***	1.04	1.03	1.06	< 0.001***

Factors associated with criminal activity included age, illicit opioid use, psychological symptoms, and MAT duration (Table 3). Men and women who were older were less likely to commit crime (OR = 0.97, 95% CI 0.96, 0.99; OR = 0.96, 95% CI 0.93, 0.98, respectively). Men and women with more opioid-positive urine drug screens were more likely to perpetuate crime (OR = 1.01, 95% CI 1.01, 1.02; OR = 1.01, 95% CI 1.00, 1.02, respectively), as were men and women who reported more psychological symptoms (OR = 1.05, 95% CI 1.03, 1.08; OR = 1.06, 95% CI 1.03, 1.09, respectively). Every year on MAT was associated with a 7% increase in the number of women engaging with criminal activity (OR = 1.07, 95% CI 1.02, 1.12; Table 3).

Older age significantly decreased the odds of reporting interpersonal conflict for men and women (OR = 0.96, 95% CI 0.95, 0.98; OR = 0.97, 95% CI 0.95, 0.98, respectively; Table 4). Men and women who endorsed higher psychological symptomatology were more likely to experience interpersonal conflict (OR = 1.03, 95% CI 1.02, 1.05; OR = 1.04, 95% CI 1.03, 1.06, respectively). Men who reported more physical symptoms and had greater opioid-positive drug screens were more likely to report interpersonal conflict (OR = 1.05, 95% CI 1.03, 1.06; OR = 1.01, 95% CI 1.00, 1.01, respectively). Women on higher treatment doses were less likely to report interpersonal conflict (OR = 0.996, 95% CI .99, 1.00). Length of time in MAT was not associated with interpersonal conflict for men or women (OR = 1.00, 95% CI 0.97, 1.03; OR = 1.00, 95% CI 0.98, 1.03).

## Discussion

This large observational study, containing 2736 patients receiving MAT for OUD, addressed the following: (1) differences in sociodemographic and clinical characteristics of men and women in treatment; (2) the SF of men and women in treatment; (3) if treatment is associated with improved SF; and (4) characteristics associated with SF.

Our results add to previous data showing that women are susceptible to a heightened burden of disease from OUD [41], with more women than men reporting unemployment, interpersonal conflict, as well as psychological and physical symptoms. The unemployment rate for women in our study was 74%, which significantly eclipsed the rate for men; both of which stagger above the rate of the Canadian general population (5% in 2018) [42]. Unemployment is a known social determinant of health; associations between unemployment and poorer health outcomes are well studied, including greater psychological distress [43], food insecurity [44], and possibly, suicide [45]. A systematic review and meta-analysis of methadone studies, many of which were conducted over 20 years ago, showed that women with OUD were at greater risk of unemployment, compared with men [27]. Our results show that the vocational landscape has not improved for women in MAT over the last two decades. A gap seems to exist in the social needs of patients in MAT; the need for integrated social and healthcare models of delivery for this large sector of the population, who are relatively young (in their 30s), is evident. Clearly, we must strive to better understand the substantial unemployment rate of MAT users, in particularly in women, through research.

Women in MAT were also more likely than men to report serious interpersonal conflict. As mentioned, interpersonal conflict was defined as verbal abuse, violence, or a major argument. That 46% of women and 35% of men endorsed conflict is alarming and likely to impact personal safety and quality of life, as well as impede recovery. Situating these findings in the pre-existing literature is challenging, given that no other studies have specifically assessed interpersonal conflict using the MAP. However, literature supporting associations, potentially causal and bidirectional in nature, between domestic abuse/violence and mental health difficulties is copious, and may be extrapolatable to our study population [46]. A review and meta-analysis found that women who had ever experienced partner violence were twice as likely to report depressive disorders, four times as likely to meet criteria for an anxiety disorder, and seven times as likely to be diagnosed with post-traumatic stress disorder (PTSD) [47]. This relationship is multidirectional and complex; as mental disorders predispose to substance use [48], substance use is a risk factor for partner violence [46], partner violence increases the risk for mental disorders [47], and mental disorders increase the perpetuation of partner violence [49]. Indeed, our study showed psychological symptoms increased the likelihood of interpersonal conflict in both men and women. There is clearly a need for future research into interventions to specifically address the perpetuation and experience of violence for people with mental health and addiction difficulties.

We found that men were more likely to report criminal activity, and also to report more episodes of crime per month, than women; results that confirm findings from older, small-sampled trials in the OUD population [27] and are consistent with statistics from the general Canadian public. Just 21% of Criminal Code offences are perpetuated by women and almost 94% of offenders in provincial custody have been identified as having a substance use problem [50]; the nature of this relationship remains murky and may be more pronounced for women [51]. Pierce et al.’s large cohort study revealed that having a history of any opioid use increased the likelihood of offending by two for men and by four for women. Compared with non-users, male and female opioid users had higher rates of criminal activity prior to opioid initiation, however, the relative risk strengthened post-opioid initiation [51]. These results are, however, limited by lack of control for important confounders, such as childhood adversity [52] and antisocial personality disorder [53].

Surprisingly, in our study, women were more likely to report criminal activity the longer they were on treatment (Table 3); every additional year on MAT was associated with a 7% increase in odds of reporting crime. Dealing drugs was the crime most often reported by women (data available upon request). This finding, coupled with the unemployment rate that remains dire for women throughout MAT, leads to one possible explanation. Women might increasingly deal drugs in attempt to mitigate persistent financial instability; however, we are unable to explore this hypothesis empirically. Results from Pierce et al. [51] highlight the potential for opioid-use prevention strategies for women in mitigating criminal activity; minimizing inappropriate prescribing and expanding purposeful work opportunities might be important approaches [54]. More work is needed to expand, and research prevention strategies for OUD, as well as better understand the role of MAT and OUD in offending, especially in women.

These results are at odds with a prior systematic review and meta-analysis of methadone studies based in China [55]. The included papers were deemed of low-quality and enrolled predominantly male cohorts. While it is difficult to draw comparisons to our study, the aforementioned meta-analysis did importantly show that longer duration of methadone was associated with reduced drug-related arrests and subtle reductions in self-reported drug-selling and sex-selling behavior.

Given the cross-sectional design of our study, the findings of increased criminality among women in MAT should be interpreted with caution. Theoretically, our findings could be explained by increased MAT retention in women with more criminal involvement. While the literature suggests the contrary, it is possible that in this case, more severe mental health problems and therefore criminal engagement may be linked to treatment seeking and study retention.

Nearly half of participants had initiated MAT within 1 year of study onset. As such, we conducted subgroup analyses to explore for differences in SF depending on treatment duration (i.e., less than, versus greater than, 1 year on MAT). Interestingly, compared to those relatively new to treatment, the group of men in treatment for more than 1 year reported less crime and conflict. It is therefore possible that men experience a delayed treatment response compared to women and that improvements in some aspects of SF may be gleaned only after 1 year.

After controlling for covariates, greater duration on MAT did not increase SF for men or for women. That being said, we did not collect data on most participants prior to treatment initiation. Thus, it is possible that participants’ SF might be substantially better than their pre-treatment state; a possibility unable to be answered by our data.

Indeed, participants in the previously mentioned NAOMI trial [21] reported a 24% increase in quality of life after 12 months of MAT. However, our study profiles may be different, as the NAOMI trial excluded some participants with severe medical and psychiatric comorbidities—confounders for both quality of life and SF. Participants in NOAMI were also treated with injectable diacetylmorphine and had psychosocial and primary care services available to them. Regardless, we present an important finding that deserves further exploration, as MAT is used as a harm reduction tool meant to improve several aspects of substance use disorder, not only the direct symptoms associated with drug effects.

Our study is the first to explore associations between SF and patient characteristics in MAT. Most noteworthy is that psychological symptoms increased the odds of poorer SF across domains (employment, criminality, conflict), for both men and women; however, the effect appears to be more profound for women. Taken in the context of the extant literature, these findings highlight the need to adequately manage distressful symptoms, particularly in women.

Continued use of illicit opioids was associated with criminal activity for both men and women in MAT. This finding affords credibility to a goal of abstinence for some people with OUD. As mentioned previously, selling drugs was the most common offence. Earlier studies have shown an association between selling and using drugs [56]. This relationship is likely bidirectional, as people might be inclined to sell drugs to support their drug use, and/or initiate their drug use because the supply is readily available; evidence from other fields certainly suggests a link between visibility and consumption [57]. It is also possible that opioids increase the risk of offending by exerting effects on cognition; the relationship between other modalities of intoxication and crime is well-documented [58].

Our study also elucidated key differences in men and women in MAT that might serve to inform future treatment programs. There is growing evidence to support tailoring addiction treatment programs to women in order to reduce potential barriers to treatment (e.g., child rearing duties); indeed, women’s only groups may have efficacy benefits [59].

### Strengths and limitations

This study is strengthened by the multisite design and large cohort of participants. To our knowledge, this is the largest study to examine sex differences in MAT, as well as the SF of men and women receiving treatment for OUD. This is also the first study, to our knowledge, to explore the impact of MAT on multiple domains of SF. While addiction studies predominantly enroll men, the relatively equivalent numbers of men and women in our study, possibly representing a more clinically relevant sample [29], is an added strength.

In every comparison in the multivariate models, most confidence intervals were very near to one. Given the potential confounders that were not controlled for, such as duration of opioid use prior to treatment, there exists possibility that, in some cases, there were no significant associations between factors and SF domains.

As mentioned, length of time on MAT was not associated with greater SF for men or for women. As this study was cross-sectional, and we also had few participants just initiating MAT (the median treatment duration was 2 years at study entry; Table 1) and therefore, there is no way to compare SF with and without MAT. It is likely that SF is different for people who have never tried MAT. It thus remains conceivable that MAT does lead to improved SF in those with a worse starting point; this hypothesis should be explored through longitudinal studies examining the impact of MAT on SF outcomes in patients newly enrolled in MAT.

On the other hand, it could be that MAT alone is not enough to improve all domains of functioning for someone with an OUD. Indeed, a recent review of the literature yielded conflicting results; some MAT trials revealed no added benefit with counseling-based interventions, and others found improvements, particularly with contingency-management interventions [60]. It should be noted that the included studies only examined the effects of non-pharmacological intervention with MAT on opioid use and treatment retention, and thus, the impact on SF remains unknown in these studies. Given the already resource-constrained healthcare systems and the unabating opioid crisis, we must also strive to predict which MAT users are most in-need and will most benefit from additional psychosocial interventions. There is, however, a dearth of literature to guide us in the development of adjunctive interventions to address SF in MAT.

The utility of SF as an outcome in MAT research may be challenging to measure and may be impacted by several other factors. One reason is the discrepancy of what SF means and what measures best capture it. A study of SF in OUD [13] measured SF using The Social Functioning Scale, a measure designed to assess social adjustment in schizophrenia [61]. As mentioned previously, this study found worse SF in people on treatment for OUD, compared to matched controls. In order to improve comparability and consistency in the literature, a standardized measure of SF in OUD should be utilized, for example, the MAP [34] or similar measures with a great degree of overlap in order to combine individual studies for future systematic reviews and meta analyses to provide a conclusive summary of evidence.

A major limitation in MAT trials, and addictions research more generally, is the inconsistency in outcomes among studies [19, 62], as well as the reliance on poorly validated outcome scales [63]. Typically, the definition of “success” in addictions treatment is arbitrarily determined and likely due, in part, to convenience [63]. Most often the outcome of choice is abstinence [64] or treatment retention [28]. There is, however, greater recognition that success may be different depending on perspective; for instance, the policy-maker may stress mortality prevention, the healthcare provider may focus on abstinence, and the patient might value employment or having a better relationship with their partner. Reducing [64] or ceasing substance use [65], minimizing psychological distress [65], and re-engaging with daytime activities [65] were recovery goals deemed important by patients with other substance use disorders.

As is the case with all studies, our findings are limited by volunteer biases, as people with worse or better SF may have been less likely to participate, and those with an un-treated OUD may have inferior SF than those enrolled in the study. Given the observational study design, we are unable to draw causal conclusions from our findings.

### Perspectives and significance

In summary, our results show that women in MAT experience worse SF than men. We also present evidence that our first-line treatment may not lead to sustained improvements in SF for people who are suffering. Indeed, women in MAT for longer reported greater engagement with criminal activity. While this study is not without limitations, we believe that the results raise important questions as to whether MAT alone can mitigate the dire SF that many people with OUD face.

## Conclusions

This study demonstrates poor SF in patients with OUD, with women disproportionally impacted. Undeniably, the poor SF of men and women involved in this study, and the lack of association between MAT duration and SF, deserves a greater exploration.

## Data Availability

The dataset used and/or analyzed during the current study are available from the corresponding author on reasonable request.
